# Latitudinal and zoo specific zeitgebers influence circadian and circannual rhythmicity of behavior in captive giant pandas (*Ailuropoda melanoleuca*)

**DOI:** 10.3389/fpsyg.2023.1188566

**Published:** 2023-09-18

**Authors:** Kristine M. Gandia, Sharon E. Kessler, Hannah M. Buchanan-Smith

**Affiliations:** Division of Psychology, Faculty of Natural Sciences, University of Stirling, Stirling, United Kingdom

**Keywords:** zeitgeber, circadian, circannual, zoo, animal welfare, behavior, breeding

## Abstract

**Introduction:**

The circadian clock influences many aspects of animal welfare including metabolism, breeding, and behavior. In most species, circadian clocks are internal clocks regulated by external environmental cues called zeitgebers. The most common zeitgebers are light/dark cycles, food, and temperature. However, within captive environments, animals can be housed at latitudes with different light/dark cycles than their natural habitat and most other zeitgebers are controlled by humans. The effects that modified zeitgebers have on captive animals’ circadian and circannual rhythmicity is largely unknown. To explore this and potential welfare implications, we measured and analyzed observational behavioral data of zoo-housed giant pandas for one year utilizing live camera footage from six zoos across the world. The worldwide distribution of the zoos gives us the unique opportunity to investigate how housing giant pandas within and outside of their natural latitudinal range can affect circadian rhythmicity and behavior.

**Methods:**

Focal sampling was completed for 11 giant pandas each month for 12 consecutive months to gain an estimate of one circannual cycle. Within each month, we estimated one daylight or 24 h cycle of activity/behavior by conducting 10-min observation sessions systemically each hour the pandas were visible.

**Results:**

Zero-inflated negative binomial mixture models found that latitude is associated with activity levels, with pandas housed outside of their natural latitudinal range displaying less activity than those within their latitudinal range. Amount of daylight, temperature minimum, and temperature range were also associated with activity cycles, potentially acting as zeitgebers. An association between sexual-related and stereotypic behavioral cycles was found, with the circannual cycles fluctuating in synchrony throughout several points in a year.

**Discussion:**

These results indicate that changes to common zeitgebers and environmental conditions can influence circadian and circannual cycles. The widespread evolution of circadian rhythms suggests an adaptive advantage to possessing one in an environment with cyclical changes, allowing species to anticipate changes in their environment and respond accordingly. Therefore, although animals are highly adaptive, creating a captive environment that mimics the environmental conditions for which the animal has evolved can encourage naturalistic cycles that ultimately aid in promoting positive welfare states and increasing chances of successful breeding and conservation.

## 1. Introduction

Providing zoo environments that promote good animal welfare is important for conservation, education, and from an ethical perspective. With the increased importance placed on captive animal welfare and the recent enforcement of welfare evaluations in zoos (World Association of Zoos and Aquariums), holistic and informative ways of evaluating welfare must be explored. One factor that could potentially influence the welfare of animals within zoos is the monitoring and management of their circadian rhythms. Circadian rhythms are the inherent, natural cycles of physical, mental, and behavioral processes that repeat roughly every 24 h. Circadian rhythms exist across the animal kingdom from birds, reptiles, mammals, amphibians, fish and arthropods, including insects (Siegel, 2008; Froy, 2011). The widespread evolution of circadian rhythms suggests an adaptive advantage to possessing one in an environment with cyclical changes, allowing species to anticipate changes in their environment and respond accordingly to maintain homeostasis. In the wild, species exhibit cycles of activity throughout a 24 h period and across the seasons. However, in captive environments, the rhythm of activity throughout the day and night and across the seasons is largely unknown as care of the animals is limited to the working day and seasonal changes in activity are not closely monitored (Brando and Buchanan-Smith, 2018). In addition, the effect of latitude on circadian rhythmicity of captive animals is not well understood. It is important to be aware of the circadian rhythms of captive species, as many aspects of their welfare, including metabolism, breeding, and behaviors, including interactions with their environment, are regulated by their circadian clock (Mellor, 2017).

Circadian rhythms operate on an approximately 24 h endogenous clock which can be entrained by external cues called zeitgebers, such as the light/dark cycle, feeding, and temperature (Buhr et al., 2010; West and Bechtold, 2015). The circadian clock regulates the clocks of peripheral tissues (liver, adipose tissue, digestive tissue, etc.) and determines the rhythms of physiological processes and behaviors (Froy, 2011; Gaddameedhi et al., 2011; Archer and Oster, 2015; West and Bechtold, 2015). Since the circadian rhythm is entrained by light, food, and temperature, natural, seasonal fluctuations in these factors cause seasonality in the circadian rhythm. Understanding the diel and annual cycles of behavior and physiology of captive species can help in gaining a view of their needs and thus inform zoo staff on measures to be taken to promote positive welfare. This approach to assessing and addressing welfare follows the 5 domains model (Mellor et al., 2020) by incorporating the assessment of nutrition, environment, health, and behavior on a diel and annual basis to interpret the affective states of species.

Species are specially adapted to the seasonal cycles of light and temperature in the habitats in which they have evolved. Intra and inter-species latitudinal clines in biological rhythmicity have been observed (Hut et al., 2013; Greenham et al., 2017; Paolucci et al., 2019). In shore birds, a latitudinal cline for incubation bout length was seen across 30 species (Bulla et al., 2016). In the parasitoid wasp *Nasonia vitripennis,* the critical photoperiod that initiates diapause (a period of suspended development) displays a latitudinal cline across populations, with populations at higher latitudes having a longer critical photoperiod (Paolucci et al., 2013). These studies demonstrate that latitude has an evolutionary effect on rhythmicity, further evidenced by latitudinal clines in the genes that regulate these rhythms (Costa et al., 1992; Hut et al., 2013; Helfrich-Förster et al., 2020). The adaptive nature of these latitudinal clines should be considered in captive settings where these adaptations are challenged by differing climatic conditions.

Changes in temperature cycles with latitude can also have a large effect on organisms, as temperature often serves as a strong zeitgeber for species that are not homeothermic vertebrates (Rensing and Ruoff, 2002). Daily cycles of temperature are particularly important for ectotherms and endothermic heterotherms, as they regulate torpor and consequently the arousal and rest periods which should be synchronized with the circadian rhythm as well as behaviors related to thermoregulation (Körtner and Geiser, 2000; Turbill et al., 2008; Abram et al., 2017). For instance, Chiara Magnone et al. (2005) found that a clock gene, Per2, was regulated by ambient temperature changes in the ruin lizard (*Podarcis sicula*). Although temperature is a weaker zeitgeber in endotherms when compared to daylight, it may enhance the amplitude of the light/dark cycle signal when in-phase, thus enhancing the entrainment signal for the circadian rhythm (Rensing and Ruoff, 2002). In addition, temperature can also be of more consequence for mammalian species which experience extreme cycles of daily temperature such as the camel (*Camelus dromedarius*), for which body temperature cycles were entrained by the ambient temperature cycles in constant light or dark conditions (El Allali et al., 2013). Understanding the changes in the cycles of light and temperature across latitudes and their effects on organisms’ circadian rhythms is important when assessing how an animal will respond to the external environment in a zoo.

A question that has not been thoroughly addressed in animal welfare research, but is of growing concern in the field, is how well captive animals can adapt to climatic and latitudinal conditions outside of their natural ranges. Because latitude establishes the extent of the plasticity of the circadian rhythm, the effects of the frequent transfer of species between zoos around the world and across latitudes must be studied and considered. Proper entrainment of the circadian clock to the external environment synchronizes animals with their environment and conspecifics, resulting in adaptive physiological, behavioral, and social functioning. In pallas cats, photoperiod stimulates gonadal activity in both males and females, synchronizing their physiological readiness to reproduce (Brown et al., 2002). However, exposure to artificial light outside of the breeding season through zoo events also stimulates gonadal activity and a subsequent refractory period which has negative effects on the ability to breed during the breeding season (Brown et al., 2002). Improper entrainment to the external environment can result in disruption or desynchrony of the circadian rhythm, which is linked to adverse health across species including reduced reproductive success, metabolic disorders, and even shortened lifespan (Hurd and Ralph, 1998; West and Bechtold, 2015; Irwin and Opp, 2017). In the nocturnal mouse lemur, exposure to light pollution changed the circadian rhythmicity of their core temperature, showing general increases in core temperature, and changed the rhythmicity of locomotor activity, with delays in the onset/offset and reduced locomotor activity during the night (Le Tallec et al., 2013).

Animals which experience seasonality may be better suited to adapt to varied latitudes as they naturally must adapt to seasonal changes. Conversely, animals within a habitat with little seasonality do not need to adjust to seasonal changes, as external cues maintain a relatively constant rhythm throughout the year. Therefore, when these animals are relocated to an area with extreme seasonality, it may be difficult for them to adapt to seasonal changes, potentially resulting in desynchrony of the circadian rhythm throughout the year or in specific seasons. To promote circadian synchrony within a zoo, it may be useful to first understand the effects that latitude has on the circadian rhythms of zoo-housed species and then mitigate the effects of extreme changes in seasonality for animals which do not experience seasonality, and to mimic certain aspects of seasonality for animals which do normally experience seasonality.

In addition to latitudinal effects on circadian rhythmicity, the zoo environment can also be influential as it is significantly different from wild environments. Though attempts are made to create enclosures which closely mimic the wild, inevitably there are many differences. In zoos, animals experience limited space, regular human presence, human-controlled schedules, artificial lighting and temperature, and enclosure designs that do not always mimic natural habitats. These factors alter the external environment and the cyclical changes experienced by the animal and have the potential to regulate the circadian rhythm. The daily rhythms of husbandry routines follow a 24 h cycle in a similar way that the circadian cycle does. Husbandry routines which include feeding, cleaning, and training sessions become a part of the external environment of the animal that is predictable in the same way light and temperature are. Therefore, in response to husbandry practices, animals may show predictable changes in their behavior and physiology.

Another ongoing question and a main goal for zoos is how to improve conservation efforts. Given the persistent threats of habitat loss and population declines in species around the world, it is essential that research continues to find innovative ways to improve the reproductive success of species in captivity. Since circadian rhythms regulate and influence many aspects of reproduction, both physiological and behavioral, understanding them can lead to improved breeding efforts. In humans, the circadian clock has been found to influence every stage of reproduction in females (Gamble et al., 2013). The circadian rhythm also determines the reproductive cycle for spontaneously and seasonally ovulating animals (Goldman, 1999). Proper entrainment to external cues and the resulting circadian rhythm has direct effects on reproductive cycles of species. Therefore, monitoring the diel and annual cycles of activity in captive species can provide insight into mating behaviors and conditions that could improve mating and reproductive success.

The welfare of captive species is paramount. However, to promote positive welfare, the welfare state of the animal and the factors affecting welfare must be well understood. The widely accepted Five Domains framework for assessing welfare addresses four physical/functional domains (nutrition, environment, physical health, and behavior) and a mental domain (Mellor et al., 2020). Circadian/circannual rhythms influence each of these domains. Therefore, monitoring and assessing rhythmicity becomes a holistic approach to assessing welfare. This holistic approach to assessing the needs of species does not only have the potential to improve conservation efforts, but can also be utilized to promote positive welfare. The inclusion of night-time activities and annual cycles in assessing welfare facilitates the understanding of the needs of the species on a daily and annual basis. This information can be used as an evidence-based approach when creating husbandry protocols, enclosure designs, and species management protocols to promote positive welfare.

To demonstrate this holistic approach and to investigate climatic/latitudinal effects on captive animal behavior, we are investigating the circadian and circannual cycles of captive giant pandas as a case study. Giant pandas have a historic natural latitudinal range of 26°N-42°N (Loucks et al., 2001). They are seasonal breeders mating between March and April (Liu et al., 2002). Females have a single, annual estrus period with mating receptivity lasting only 1–3 days (Schaller, 1985). The mating strategies involve females and males synchronizing multimodal signaling behavior around the estrus cycle with social/sexual behaviors increasing during this period (Owen et al., 2013) and mate choice increasing the likelihood of successful breeding (Martin-Wintle et al., 2015). Therefore, although hormonal data was not available for this study, we can still demonstrate how understanding the circadian rhythmicity of these sexual-related behaviors can aid zoo staff in identifying when their pandas are receptive for breeding and if behaviors are desynchronized with estrus if zoos then pair the behavioral data with hormonal data.

Giant pandas are also a migratory species, following their food source of bamboo with a preference for nutritious shoots that emerge in Spring (Wang et al., 2010). They initiate migration from middle April to early June (coinciding with the breeding season), migrating within several days to their Summer range and returning over several weeks from early September to October (Liu et al., 2002). In the wild, giant pandas exhibit three peaks in activity throughout the diel cycle and a fluctuation in activity levels throughout the year, with a peak in June (Zhang et al., 2015). Giant pandas being such a specialized species, evolving over time to eat bamboo and adapting their behavior for this food source, makes them an ideal animal for this study as they would presumably need to be well synchronized to their environment to sustain their lifestyle. Giant pandas are also ideal for this study because they are a charismatic species and have large popularity worldwide. This means that they have many available webcams for observation across different latitudes that allow us to study latitudinal effects on cycles and show a proof of concept of how monitoring animals on cameras can be very informative while being non-invasive. In addition, pandas are a vulnerable species that are notoriously difficult to breed in captivity. Though large improvements in the captive breeding of pandas have been made, with mate choice being identified as a key factor in successful breeding (Martin-Wintle et al., 2015), further understanding of how pandas synchronize their breeding behavior could greatly improve conservation efforts for successful breeding of captive pandas.

In this study, we recorded circadian cycles for one year for captive giant pandas at latitudes that are matched and mismatched to their natural latitudinal range. We investigated the latitudinal effects on behavioral cycles and how the information from these cycles can be used to understand the environmental, behavioral and social needs of pandas. We hypothesize that there will be differences in behavioral cycles across latitudes due to the differences in the cycles of the external zeitgebers they are exposed to. We also hypothesize that zoo environments will have additional zoo specific zeitgebers like husbandry routines that may synchronize with behavioral cycles. Our study cannot determine causality, but finding relationships between behavioral cycles and external factors can help inform zoos on how they can better provide for the needs of their animals, and can give insight on the husbandry and environmental factors that may promote the positive welfare states they want to see in their animals.

## 2. Materials and methods

### 2.1. Ethics statement

We received ethical approval for this study from the University of Stirling Animal Welfare Ethics Review Body (protocol #2084 1,591; 30/04/2020). The study also received support from the Association of Zoos and Aquariums, Giant Panda Species Survival Plan (05/08/2020). In addition, we submitted research applications to all zoos involved and received approval from the participating zoo’s administrations. We also sent a voluntary questionnaire to keepers asking information on (1) panda identities, (2) cameras, (3) enclosure design, (4) artificial lighting, (5) feeding husbandry and (6) breeding opportunities. Three zoos provided some or all of the requested information. The identifying data for the zoos and pandas has been anonymized.

### 2.2. Study animal selection

The inclusion criteria for a giant panda was whether the panda was in a zoo that had a publicly accessible web camera or a surveillance system that we could be allowed access to. If the zoos approved the use of their cameras and the pandas were consistently in view, the pandas remained in the study. As a charismatic species with large public interest, giant panda web cameras are very popular, making the species ideal for this study because we were able to select individuals from around the world. We selected 11 giant pandas (6 females, 5 males) from five zoos (Table 1). We identified whether a panda was housed in a latitude within or outside of the giant panda historic natural latitudinal range (26°N-42°N; Loucks et al., 2001), with the southern hemisphere’s mirrored latitudes (26°S-42°S) counting as a matched latitude. Zoos A and C are located at latitudes that match the natural range of giant pandas while Zoos B, D, and E are located at latitudes above the natural range toward the poles. There were no zoos located outside of the natural range toward the equator. We also identified the pandas by sex and age group, with sub-adults being individuals aged 6 years or younger. This age was decided based on the life stage in which giant pandas reach sexual maturity (Liu et al., 2006). All cameras were accessible 24 h, however, cameras for 6 individuals did not have night-vision, so observations for these individuals were limited to daylight hours (Table 1). The remaining 5 individuals had data collected on a 24 h basis.

**Table 1:** Information on the zoo latitude, study animals, husbandry, indoor lighting, and temperature.

Zoo	Zoo latitude	Panda	Sex	Age group	Breeding opportunity	Camera visibility	Indoor lighting	Indoor temperature
A	Match	1	F	Adult	Around March; either natural or artificial insemination	Daylight	Unknown	Unknown
		2	M	Adult				
B	Mismatch	3	F	Sub-adult	Breeding pair, but unknown timing and method	24 h	Unknown	Unknown
		4	M	Sub-adult				
C	Match	5a	F	Sub-adult	None	Daylight	Natural + Artificial Turned on when needed	15–21\u00B0C year-round
		5b	F	Sub-adult				
		6a	F	Adult	Post-reproductive			
		6b	M	Adult				
D	Mismatch	7	F	Sub-adult	Around March; natural	24 h	Natural + Artificial Scheduled with dimming mimicking light in Chengdu, China	Manually controlled
		8	M	Adult				
E	Mismatch	9	M	Adult	Castrated for medical reasons	24 h	Natural + artificial Turned on/off at beginning and end of day	Mimic outdoor temperature. Heaters that are kept between 12–16\u00B0C giving panda choice.

Within Zoo C, sub-adults could be distinguished from adults, but individuals could not be identified. Therefore, these individuals are listed as 5a, 5b, 6a, and 6b. Individuals with the same number had their data combined and the sex was omitted in analysis for individuals 6a and 6b. Zoo E also housed a female panda whose data could not be collected for the study because her footage was not accessible. Zoos A and C are located at latitudes that match the natural range of giant pandas while Zoos B, D, and E are located at latitudes above the natural range toward the poles. Breeding opportunity refers to when pandas are normally given opportunities by keepers to breed naturally or through artificial methods.

### 2.3. Behavior observations

Behavioral observations were completed using the ZooMonitor application (Ross et al., 2016). The ethogram was designed to include most of the behavioral repertoire of giant pandas, covering behaviors that indicate positive, neutral, and negative affective states (Supplementary Table 1).

**Figure 1:**
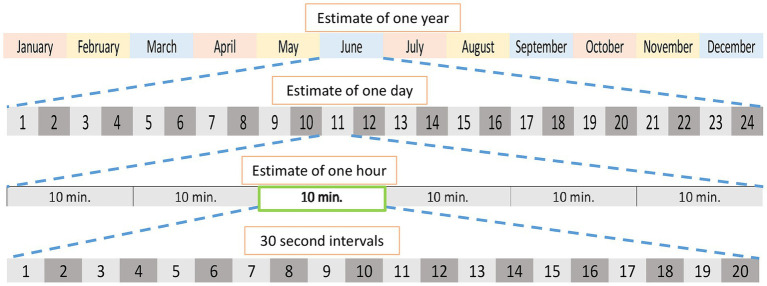
Representation of the sampling method to obtain estimates of circadian cycles and a circannual cycle. Each month, observations began on the 10th of the month and continued through the end of the month until one 10-min session was completed for each hour of the day. For pandas observed on cameras without night vision, observations continued until a 10-min session was completed for each daylight hour. Completion of 10-min sessions depended on the availability of an observer and the visibility of the panda on camera. Figure reproduced from Gandia et al. (2023).

All observations were made through web cameras from December 2020 – November 2021. Focal sampling was completed for each giant panda using 10-min sessions with 30-s intervals every hour to gain an estimate of behavior in that hour. Each month, data collection began on the 10th day and continued through the end of the month until one daylight or one 24 h cycle was recorded (dependent on the night-vision of the camera) for each panda. The 10-min sessions were completed depending on the availability of an observer and the presence of the giant panda on camera. A graphical representation of the sampling method can be seen in Figure 1.

Along with behavior, we also recorded the following at each interval: whether the panda was in their indoor or outdoor enclosure and whether the camera was displaying in color or black and white (due to ambient light becoming dark). Vocalizations were not recorded as some cameras did not have audio. Whether the panda was out of sight was also recorded, and a session was only saved if the panda was in sight for 60% of intervals (12/20 intervals) to provide representative data for analysis. Sessions with more than 8/20 out of sights were deleted and redone.

In total, 13 observers assisted in data collection throughout the data collection period. To produce data that would be used for analysis, observers had to pass reliability testing. Since testing reliability purely from live observations results in many ethogram behaviors not being evaluated (Wark et al., 2021), we designed our reliability testing with two stages aiming to cover all ethogram behaviors. The first stage was an ethogram quiz for which the observer had to receive >80%. The second stage was inter-observer reliability using a combination of compilations of short video clips of all behaviors listed in the ethogram and 10-min recordings from the study pandas mimicking the way observation sessions would be completed using the web cameras and ZooMonitor. For the short video clips, beeps were placed at variable intervals so the full repertoire of pandas was covered, and indicated when to record a behavior. For the 10-min recordings, observers used ZooMonitor and the 30 s intervals to record. Observers had three attempts (each attempt had different videos or beeps were changed) to match at least 75% of the recordings from the lead investigator to pass this final stage. The 75% agreement threshold was based on those generally accepted for reliability (Graham et al., 2012). Each attempt contained 8–9 video clips with 4–5 beeps each, and 3–4 10-min recordings with 30-s intervals.

### 2.4. Analyses

#### 2.4.1. Variables

Two of the main goals of zoos are to conserve species and promote positive welfare of their animals. Therefore, in order to investigate ways in which zoos can further these goals, we focused our analysis on general activity, sexual-related behavior, and stereotypic/abnormal behavior. Determining how zeitgebers potentially regulate or synchronize with these behavior categories can help us understand how to create environments that are more conducive to the expression of positive and sexual-related behavior. Each behavior category- activity, sexual-related, and stereotypic/abnormal- was calculated by adding the counts by 10-min session of respective behaviors listed in Table 2. We modeled activity, sexual-related behavior, and stereotypic/abnormal behavior in separate models against several predictor variables. The activity category (all behaviors except for resting/sleeping) is used since circadian rhythms are often described as patterns of active/inactive states. The predictor variables were zoo latitude as described in Table 1 (categorical with two levels: match and mismatch), temperature minimum, temperature range, amount of daylight in hours, season, and hour of the day. Temperature measures and daylight were recorded from a weather website (timeanddate.com). The location of the zoo was searched and the maximum/minimum temperature and number of daylight hours recorded from the same date as the observation. In addition, age group and sex were included in the model as controls.

**Table 2:** Behaviors combined for the behavior categories analyzed.

Behavior category	Combined behaviors
Activity	All behaviors except for resting/sleeping.
Sexual-related	Anogenital rubbing, sexual, show interest, and scent-anoint.
Stereotypic/Abnormal	Pace, bipedal standing, self-mutilation, cage climb, regurgitation, pirouette, and head-toss.

See Supplementary Table 1 for full list and definition of behaviors.

#### 2.4.2. Zero-inflation negative binomial modeling

It is common for ecological data to have a high amount of zero values resulting in zero-inflation that causes significant biases in analysis because the fit regression becomes flat (Martin et al., 2005; Fávero et al., 2021). These zeros are either ‘true zeros’ or ‘false zeros.’ With behavioral data, true zeros are observed from individuals that never display a behavior or because a behavior is not constantly displayed or rare. These zeros are also called structural zeros. False zeros occur from sampling error, if a behavior is not displayed within the sampling period or if a behavior is miscoded. These zeros are also known as sample zeros.

Within our data, each of our response variables had a very high percentage of zeros: activity, 46.3%; sexual-related, 97.2%; stereotypic/abnormal, 91.9%. Our data will have both true/structural and false/sample zeros, but mainly zero-inflation due to true/structural zeros, which results in overdispersion (Martin et al., 2005). Therefore, we needed a model that worked for count rate data (counts of behavior within a 10-min session) and would account for zero-inflation and the resulting overdispersion. Negative binomial models are count models that have a parameter that allows for overdispersion (Martin et al., 2005; Lindén and Mäntyniemi, 2011). The most appropriate model considering the qualities of our data was the zero-inflation negative binomial (ZINB) mixture model. To conduct this analysis, we used the R package *glmmTMB* (Brooks et al., 2017). In *glmmTMB*, zero-inflated GLMMs have three components: a model for the conditional mean (Negative binomial in our study), a model for zero-inflation, and a dispersion model. The conditional mean and dispersion models analyze positive values using log links. The zero-inflation model describes the probability of observing a true/structural zero that is not generated by the conditional model. The values within the zero-inflation model are constrained between 0 and 1 by applying a logit link (Brooks et al., 2017, 2019; Fávero et al., 2021). The overall fit of the ZINB mixture model is determined by all three components. Therefore, when interpreting the results, we must consider the results of all three models. The interpretation of the coefficients for the count model and zero-inflation model are different. A positive coefficient in the count model indicates an increase in the response with an increase in a continuous predictor or in that level of the categorical predictor compared to the other levels. In contrast, a positive coefficient in the zero-inflation model indicates that a structural zero in the response is more likely with an increase in a continuous predictor or in that level of the categorical predictor compared to the other levels.

For each behavior category ZINB mixture model, the zero-inflation model was the same as the conditional model which included pandas nested within zoos. The categorical variables of season, age group and sex were coded within the model using contrast sums. Therefore, each level of the variable was compared against a grand mean within the model which was the mean of the response variable means at each level of the categorical variable. Due to our limited sample size, we ran the models using Restricted maximum likelihood rather than Maximum likelihood. This is an iterative process and the final model that is presented is the one with the best estimation. We did not standardize time out of sight by converting the counts to decimal rates per time in sight because the models require count data and because, among all sessions, the mean time in sight was 95.6% and the median was 100%. *Post hoc* analysis of pairwise comparisons of estimated marginal means (least-squares means) were conducted on the season variable using the R package *emmeans* (Lenth, 2023) to determine any significant differences between variable levels. Multiple comparisons were controlled using the Tukey method. Test-wide alpha was set at 0.05.

For the stereotypic/abnormal behavior ZINB model we added sexual-related behaviors as a predictor because a study on 101 captive giant pandas by Martin et al. (2020) found an association between stereotypic behavior and reproductive performance. However, since stereotypic/abnormal behaviors are not seen in wild giant pandas, and therefore would not predict sexual behavior in the wild, we did not include stereotypic/abnormal behaviors as a predictor in the sexual-related behavior ZINB model. In addition, *ggplot* allows for two kinds of negative binomial models, one which models the count data with linear regression and another which uses quadratic. For the stereotypic/abnormal behavior model we used the quadratic negative binomial because it reduced the dispersion 10-fold, implying a better fit.

#### 2.4.3. Wavelet coherence analysis

In order to extrapolate more information on how behaviors and zeitgebers synchronize/desynchronize throughout the year, we used continuous wavelet transform coherence analyses. Wavelet transform is a time series analysis where a signal is transformed into a wave with zero mean that is expanded and localized in both frequency and time. This allows for the detection of periodic patterns of a time series in both time and frequency domains while controlling for random background noise in the signal. Wavelet transform is useful for analyzing localized intermittent oscillations in a single time series, but we can also use a wavelet coherence analysis to determine how two time series are related to each other. With wavelet coherence analysis we can examine whether regions in time frequency space with similar high power have a sustained phase relationship, possibly suggesting a relationship between the signals (Grinsted et al., 2004). Essentially, we can determine areas of correlation between the two wavelets.

This type of analysis is ideal for our questions addressing the relationships between zeitgebers and behaviors. It allows us to identify times of the year in which the zeitgeber and behavior are correlated and determine the kind of phase relationships and the implications for how the zeitgeber potentially regulates or synchronizes with that behavior. We can also determine whether these relationships would change based on the latitudinal location. For our analysis, we conducted a wavelet coherence analysis for the continuous predictor variables that came out significant in the ZINB models so that we could extrapolate more information on the kind of relationship between the predictor and respective behavior category.

To conduct the analysis, we used the MatLab Wavelet Toolbox developed by Grinsted et al. (2004). Our data are non-stationary, so we used a continuous wavelet analysis with the Morlet wavelet and a scale resolution of 10 scales per octave, as suggested by Grinsted et al. (2004), since these settings provide a good balance between time and frequency localization. Our sampling period was set as Δt = 1 h. We also decided to conduct continuous wavelet transform and wavelet coherence analysis because these analyses were used by Zhang et al. (2017) to address similar questions in wild giant pandas on activity data recorded using radio collars. We followed their analyses as closely as possible so that our data could be compared to the results found in wild pandas. However, because of our sampling method, our behavior signals are constructed of consecutive, representative 24 h periods in each month. Therefore, we could only determine the patterns of circadian cycles and the annual cycle of those circadian cycles but could not infer anything about rhythms with a month-long period.

#### 2.4.4. Indoor vs. outdoor activity levels and budgets

Most captive animals have both indoor and outdoor enclosures. Zeitgebers tend to be controlled for indoor enclosures (i.e., temperature and artificial lighting). However, for outdoor enclosures, the zeitgebers are those for the latitude which the zoo is located. Because of this, we wanted to examine how time outdoors is associated with activity levels. To provide more context to this relationship, we determined the activity budgets while indoors versus outdoors and looked at the difference between the latitude groups since pandas at mismatched latitudes would presumably experience outdoor external factors for which they are not adapted to. We conducted Welch’s paired sample t-tests to compare the levels of locomotion, resting/sleeping, feeding, and stereotypic/abnormal behavior between the indoor and outdoor enclosures within each latitude category (matched/mismatched).

## 3. Results

Model summaries for the three behavior categories can be seen in Table 3. Each behavior category did indeed display overdispersion. Each of the behavior category ZINB mixed models had significant coefficients in either the count model or the zero-inflation model, or both. Below we will describe the results for both components, addressing the count model and then the zero-inflation model. Our interpretation of the data in the Figures is usually based on both visual inspection of the overlapping confidence intervals combined with which variables came out as significant in the models.

**Table 3:** Summary of ZINB models for activity, sexual-related behavior, and stereotypic/abnormal behavior.

Behavior category	Regression type	Iterations	AIC	BIC	Df(residual)	Dispersion parameter
Activity	Linear	87	9884.4	10045.9	1921	3.59
Sexual-related	Linear	49	N/A	N/A	1921	1.62
Stereotypic/Abnormal	Quadratic	65	1979.4	2152.0	1920	1.14

N/A is indicated where a score was not produced because of model convergence.

### 3.1. ZINB model results for activity

Within the count model, the negative coefficients for temperature minimum (−0.007, z = −1.970, *p* = 0.049) and temperature range (−0.014, z = −2.955, *p* = 0.003) suggest a decrease in activity with an increase in both the temperature minimum and temperature range for the day. The trend toward significance for the positive coefficient for daylight (0.017, z = 1.828, *p* = 0.068) indicates a potential, slight increase in activity with an increase in amount of daylight. A trend toward significance was also seen for activity at mismatched latitudes (−0.139, z = −1.834, *p* = 0.067) and could suggest a potential, slight decrease in activity at mismatched latitudes compared to activity displayed at matched latitudes.

Within the zero-inflation component, latitude was significant, with the positive mismatch coefficient (1.058, z = 6.082, *p* < 0.001) suggesting that pandas at a mismatched latitude are more likely to have a true/structural zero for activity when compared to pandas at matched latitudes. In other words, pandas at mismatched latitudes generally show more sporadic activity than pandas in matched latitudes. The negative coefficient for Spring (−0.402, z = −2.052, p = 0.04) indicates that during this season, it is less likely for a panda to display a true/structural zero in activity. This means that during Spring pandas are more likely to be consistently active. Conversely, the positive coefficient for Autumn (0.220, z = 2.104, *p* = 0.035) suggests that pandas are more likely to show a true zero in activity during Autumn. Hour of day was also significant (−0.018, z = −2.265, *p* = 0.024). *Post hoc* comparisons did not reveal any significant differences in activity between seasons.

**Figure 2:**
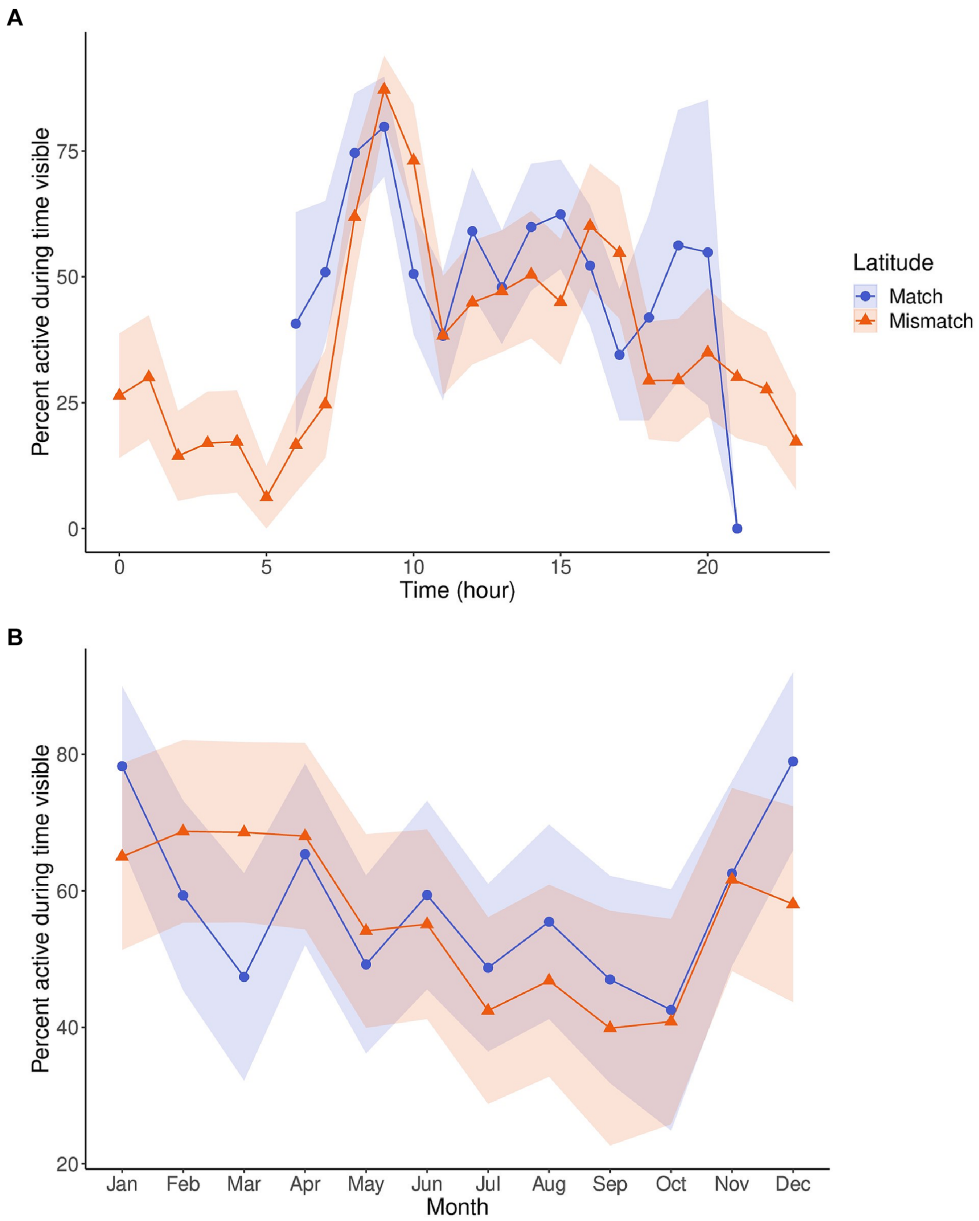
Line plots displaying the circadian cycles **(A)** and circannual cycles **(B)** of activity for pandas in matched (blue, *n* = 6) and mismatched (orange, *n* = 5) latitudes. Activity is displayed as percentage of time active while in sight, controlling for time out of sight, and was averaged by hour **(A)** or month **(B)**. The shaded regions are the 95% confidence intervals for the activity. The circadian cycle for pandas at matched latitudes is only for daylight hours, as these pandas had cameras without night vision, and therefore could not be recorded. The circannual cycles displayed are from a 9-h daylight subset of data so that it could be comparable between the two latitude categories.

Figure 2 depicts the circadian and circannual cycles of activity by latitude. We found similar circadian patterns of activity between the matched and mismatched groups despite their latitudinal difference. Likewise, the circannual cycle of activity between the two groups are very similar and only diverge between December and March, which includes part of the breeding season in March and April (Liu et al., 2002).

### 3.2. Activity wavelet coherence analyses

**Figure 3:**
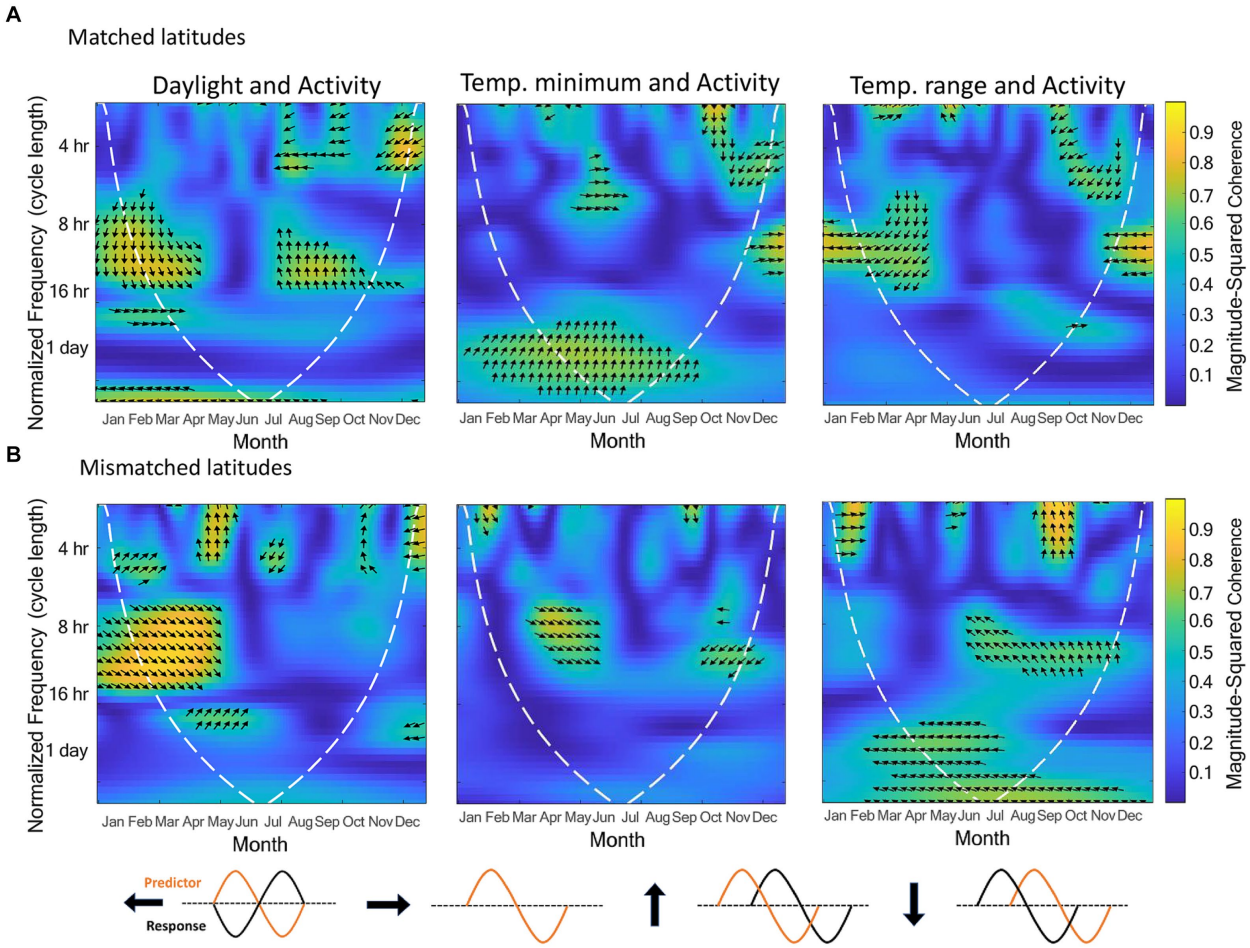
Wavelet coherence analyses between activity and daylight, temperature minimum, and temperature range, respectively. The top row shows these coherence analyses for pandas in matched latitudes (*n* = 6), and the bottom row for pandas in mismatched latitudes (*n* = 5). The data entered for the analyses were a 9-h subset of daylight data so that comparisons can be made between the two latitude categories. The dashed white line indicates a 5% significance level. The *x* axis is the time of year in months, the *y*-axis is the normalized frequency between the two signals, and the color represents the strength of the correlation (yellow high, dark blue low, scale on right hand side). The kind of phase relationship between the signals are noted by the arrows (refer to arrow key).

**Figure 4:**
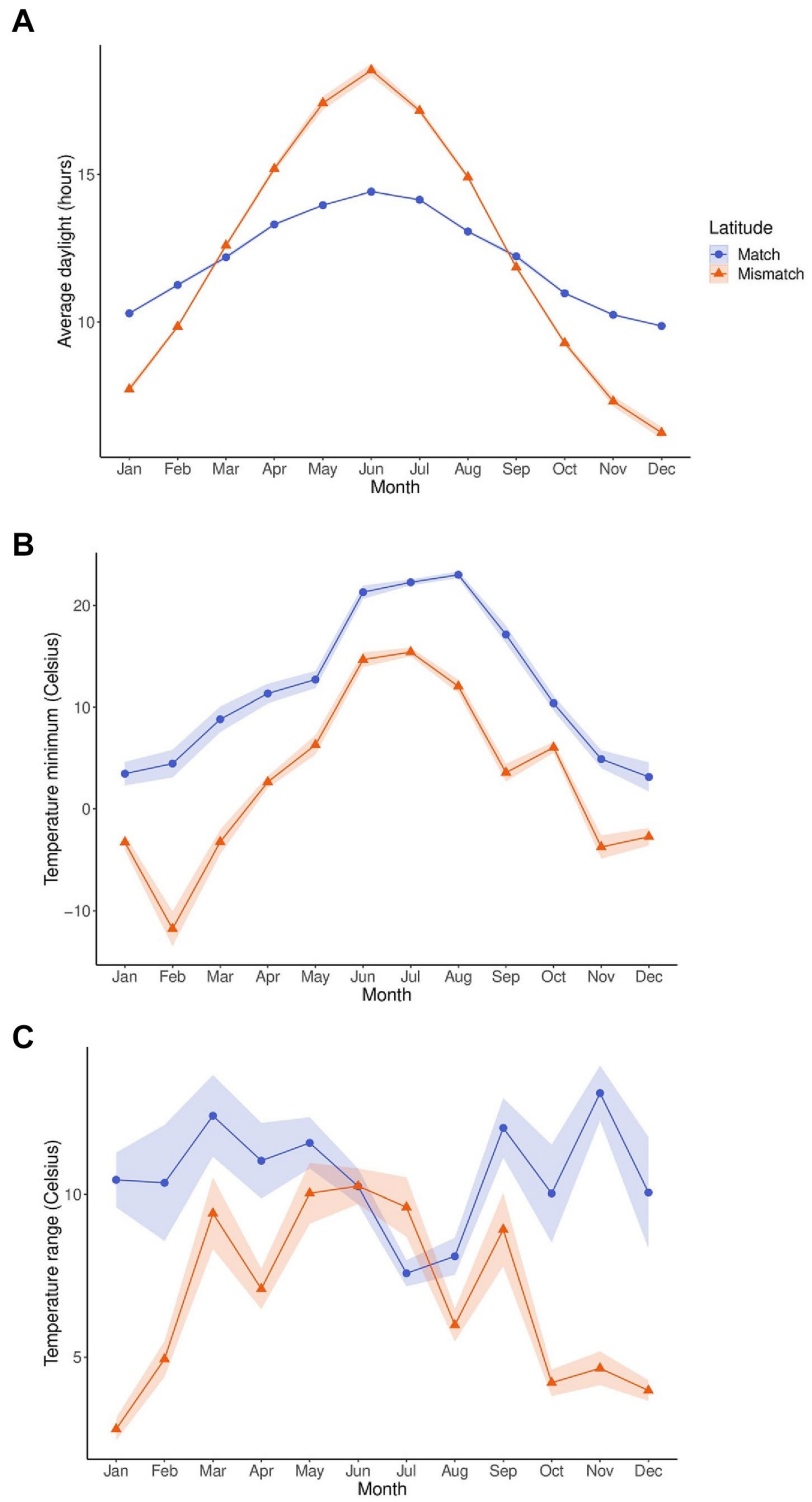
Annual cycles of average daylight **(A)**, temperature minimum **(B)**, and temperature range **(C)** between the zoos at matched (blue, *n* = 6) and mismatched (orange, *n* = 5) latitudes. The measurements were averaged by month. The shaded regions are the 95% confidence intervals of the measurements.

Since temperature range and temperature minimum were significant, and daylight near significance in the count model for activity, and latitude near significance in the count model and significant in the zero-inflation component, we also analyzed these three zeitgeber measures by latitude using wavelet coherence (Figure 3). Matched and mismatched latitudes had different circannual cycles of these zeitgeber measures (Figure 4). There was a much larger change in daylight throughout the year at the mismatched latitudes (Δh ≈ 15) when compared to matched latitudes (Δh ≈ 5). The temperature minimum was consistently lower throughout the year at mismatched latitudes compared to the temperature minimum at matched latitudes. The temperature range throughout the year between the latitudes seemed to be completely out of phase with each other.

The wavelet coherence analyses (Figure 3) show how the phase relationships between activity and the three zeitgebers differed between the latitude locations. For the coherence between daylight and activity, both latitudes showed similar coherence in Spring at the normalized cycle lengths of 8-16 h with the activity signal leading, but in Autumn, matched latitudes showed a lagging activity signal, while mismatched latitudes did not show much coherence at all. In the coherence analysis for activity and temperature minimum, the two latitude groups displayed some similar coherence between May and June around the 8 h cycle length with the signals being nearly in phase with each other, and a small similarity from September to October with activity shifting from leading to nearly out of phase. However, around the 1-day cycle length, the matched latitude displayed a consistent phase relationship that was not present for mismatched latitudes between April and August, with the activity signal leading. The coherence analyses for activity and temperature showed no similarities between the latitudes. Coherence occurred at different cycle lengths and times of year, or with differing phase relationships.

### 3.3. Outdoor vs. indoor activity

**Figure 5:**
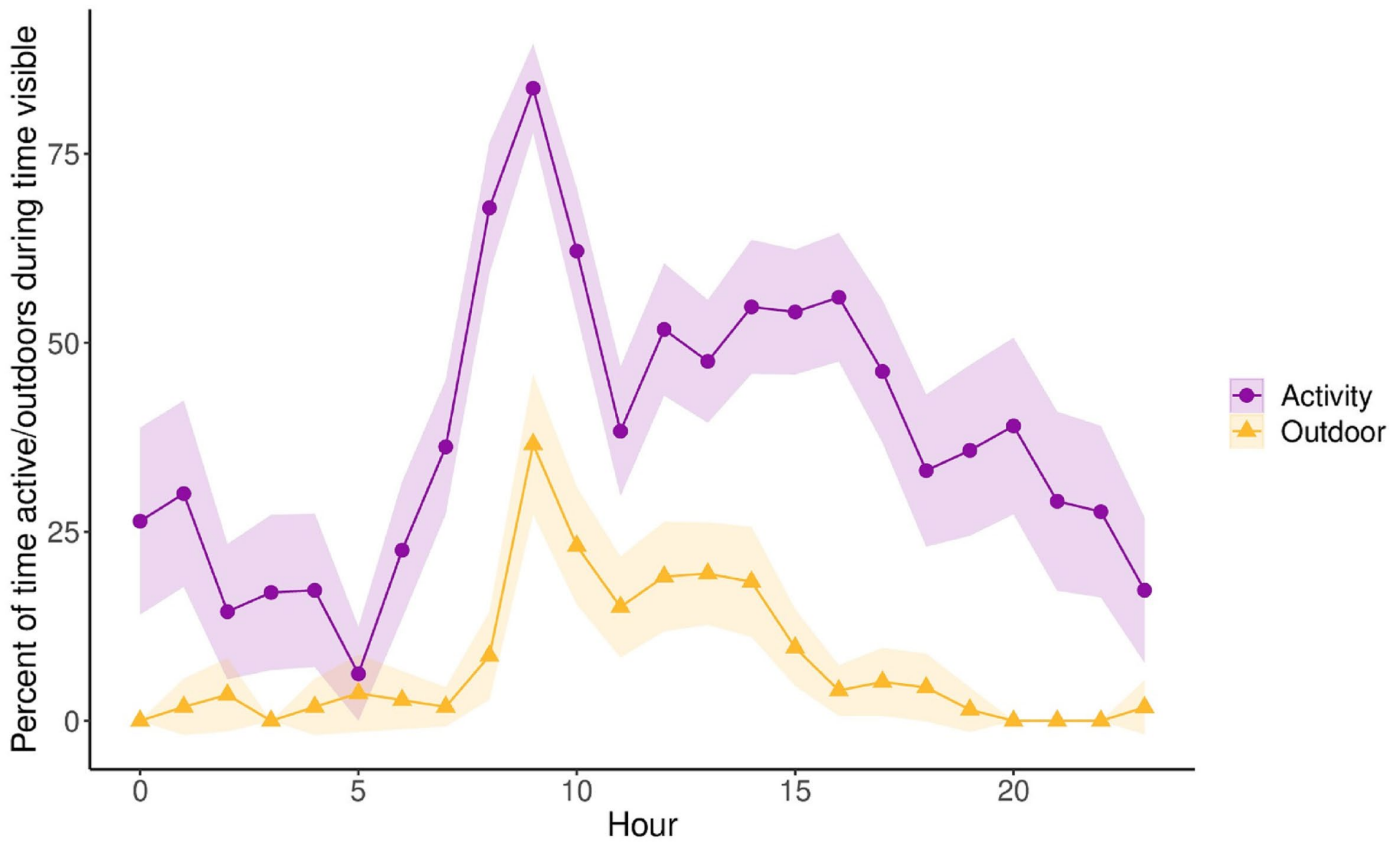
Percentage of time active (purple) and outdoors (yellow) throughout the circadian cycle for all pandas (*n* = 11). Measurements are displayed as percentage of time while in sight, controlling for time out of sight, and averaged by hour. Shaded regions are 95% confidence intervals. The percentage outdoors is opposite to the percentage of time indoors. Therefore, a measurement of 0% time outdoors represents 100% time indoors and vice versa.

**Figure 6:**
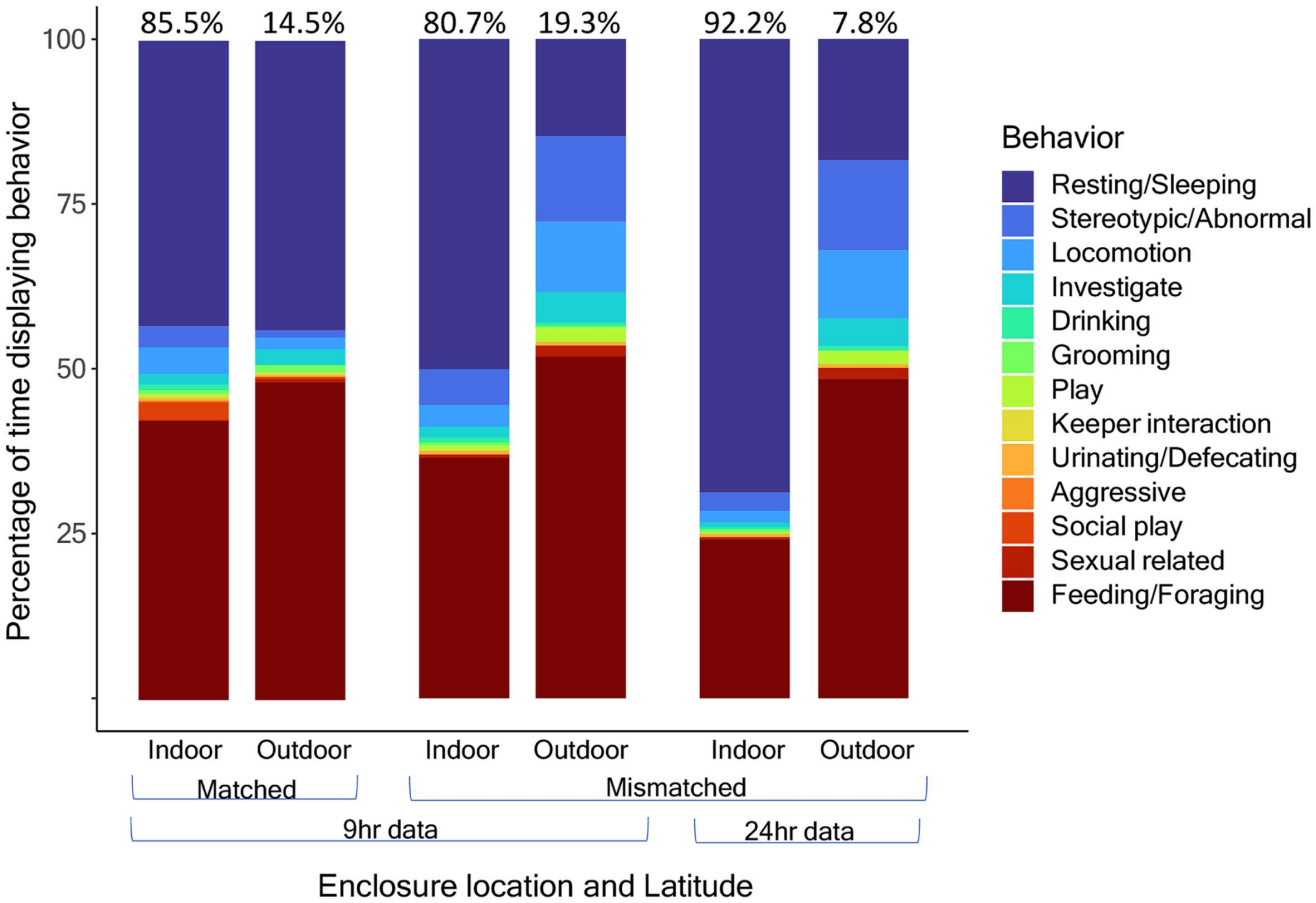
Activity budgets in percentage for indoor and outdoor enclosure space compared between pandas at matched (*n* = 6) and mismatched (*n* = 4) latitudes. Panda 9 is excluded from this analysis because cameras were fixed on the indoor enclosure most of the year. Data are proportions of time represented as percentages out of 100%, not counts. For pandas in mismatched latitudes, activity budgets are further compared between the 9-h daylight subset of data, for comparison with the matched latitudes, and the full 24 h of data. The color indicates the behavior. Percentages at the top of the bars indicate the total percentage of time spent indoors or outdoors.

When comparing the circadian cycles of activity levels and percentage of time outdoors, we found that activity levels coincide almost perfectly with the amount of time outdoors (Figure 5). For the activity budget comparison indoors vs. outdoors and by latitude, we found that pandas at matched latitudes displayed very similar activity budgets both indoors and outdoors with no significant differences in locomotor, resting/sleeping, feeding and stereotypic/abnormal behavior between the indoor and outdoor enclosures (Figure 6). Meanwhile, pandas at mismatched latitudes displayed a significantly higher proportion of locomotor activity in the outdoor enclosures (t = −2.157, df = 56, *p* = 0.035).

### 3.4. ZINB model results for sexual-related and stereotypic/abnormal behavior

The model for sexual-related behavior displayed a model convergence warning, which indicated that the best fitting model was likely not found, and therefore, the AIC and BIC scores were not produced. However, the model was run several times and produced the same values within the count and zero-inflation model. In the count model for sexual-related behavior, the positive coefficient for Spring (1.634, *z* = 3.157, *p* = 0.002) suggests an increase in sexual-related behavior during this season compared to the grand mean of all seasons. This increase coincides with the breeding season. Conversely, the negative coefficient for Autumn (−1.412, *z* = −2.158, *p* = 0.031) and potentially significant negative coefficient for Winter (−1.105, z = −1.843, *p* = 0.065) suggest decreases in these seasons compared to the grand mean for all seasons. *Post hoc* comparisons confirm these patterns showing Spring having significantly higher sexual-related behaviors than Autumn (3.046, z.ratio = 2.908, *p* = 0.019) and Winter (2.857, z.ratio = 2.857, *p* = 0.022). Hour of day (0.273, *z* = 5.761, *p* < 0.001) was also significant. Within the zero-inflation component, the hour of day coefficient (0.612, *z* = 4.392, p < 0.001) was also significant. The positive coefficients for adults (1.438, *z* = 2.307, *p* = 0.021) and females (2.579, z = 2.885, *p* = 0.004) suggest that these groups are more likely to show true zeros for sexual-related behavior.

In the count model for stereotypic/abnormal behavior, the positive coefficient for Spring (0.494, *z* = 2.876, *p* = 0.004) indicates an increase in stereotypic/abnormal behavior compared to the grand mean for all seasons, coinciding with the breeding season. However, for Winter (−0.587, *z* = −2.384, *p* = 0.017), stereotypic/abnormal behavior decreased in comparison to the grand mean of all seasons. *Post hoc* comparisons confirm this result with stereotypic/abnormal behavior being significantly higher in Spring compared to Winter (1.082, z.ratio = 3.080, *p* = 0.011). In addition, sexual-related behavior showed a decrease as stereotypic/abnormal behavior increased (−0.506, *z* = −4.381, *p* < 0.001). Together, these results follow predictions that stereotypic/abnormal behaviors are related to sexual-related behaviors since they increase in the breeding season and show an inverse relationship to sexual-related behaviors. The latitude mismatch coefficient (1.127, *z* = 1.845, *p* = 0.065) trended toward significance in the count model, suggesting potentially higher levels of stereotypic behaviors seen for pandas at mismatched latitudes compared to those at matched latitudes. The coefficient for males (−0.398, *z* = −2.173, *p* = 0.030) was also significant in the count model. Within the zero-inflation component, the positive latitude mismatch coefficient (1.251, *z* = 2.640, *p* = 0.008) indicates that those pandas located in mismatched latitudes are more likely to have a structural zero for stereotypic behavior, perhaps displaying them more sporadically but at higher levels, as the count model would suggest. The negative coefficients for Spring (−0.436, *z* = −2.345, *p* = 0.019) suggests that pandas are generally more likely to show positive values for stereotypic/abnormal behavior in the Spring compared to other seasons. The negative coefficient for sexual-related behavior (−2.622, *z* = −4.500, *p* < 0.001) follows predictions that with an increase in sexual-related behavior, it becomes more likely that the panda will display some level of stereotypic/abnormal behavior. Hour was also significant within the zero-inflation model (0.034, *z* = 1.993, *p* = 0.046).

**Figure 7:**
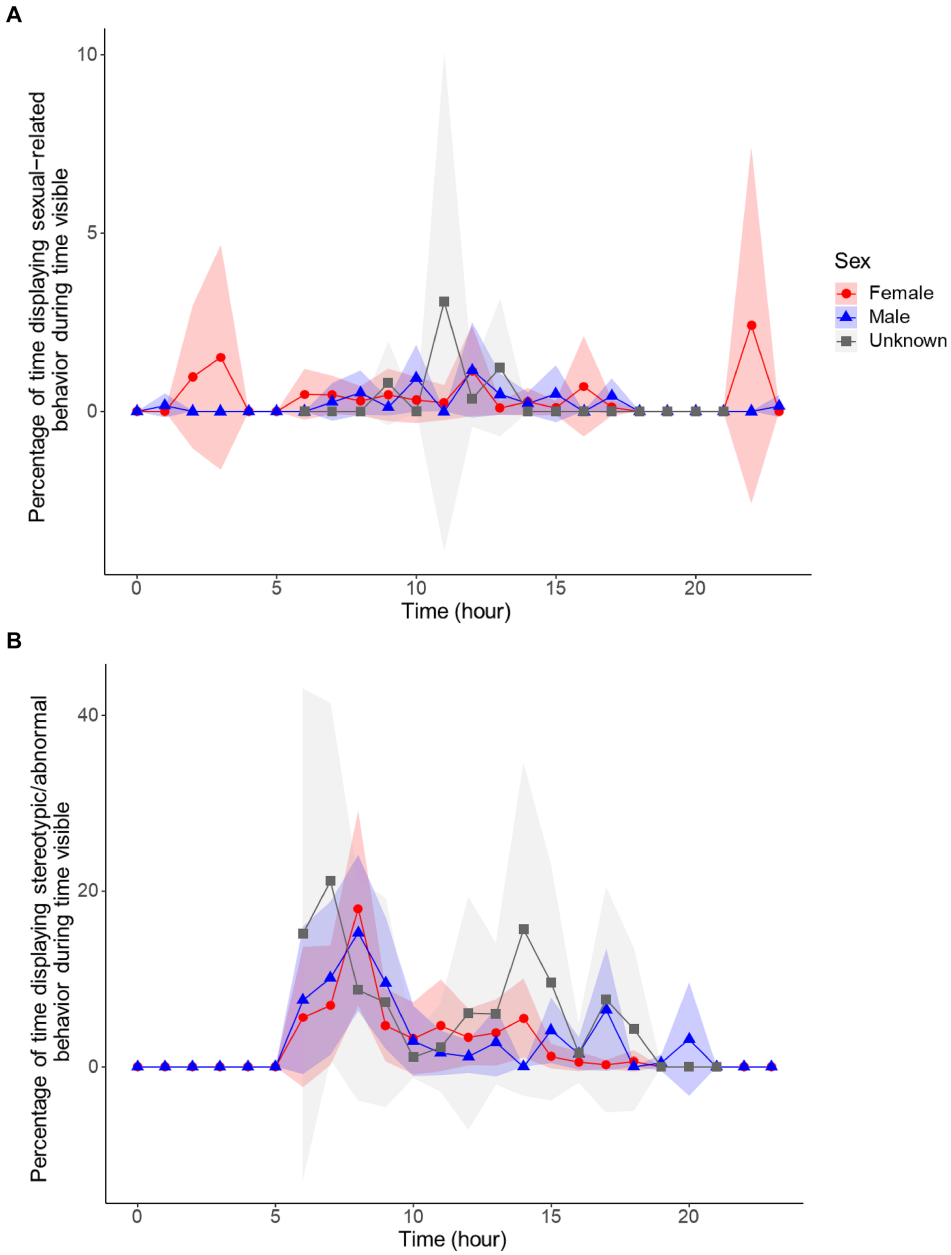
Circadian cycles of females (red, *n* = 5), males (blue, *n* = 4), and unknown sex (*n* = 2) for sexual-related **(A)** and stereotypic/abnormal **(B)** behavior. Behaviors are percentages of time in sight, controlling for time out of sight, and averaged by hour. Shaded regions are the 95% confidence intervals.

**Figure 8:**
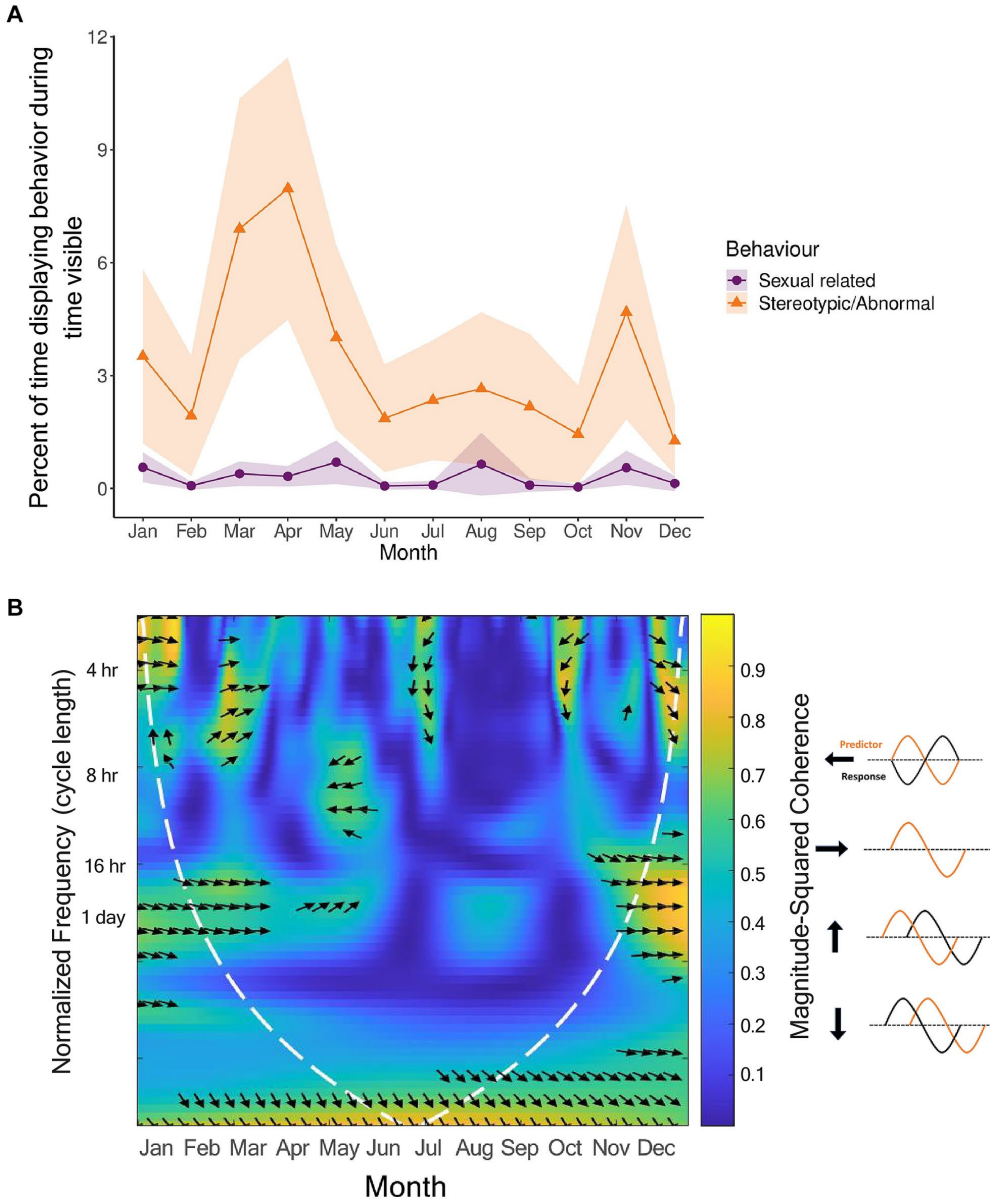
Relationship between sexual-related (purple) and stereotypic/abnormal (orange) behaviors for all pandas (*n* = 11). Circannual cycles **(A)** for the behaviors are percentages of time in sight, controlling for time out of sight, and averaged by hour. Shaded regions are the 95% confidence intervals. The data for the wavelet coherence **(B)** are the averaged signals for all pandas using the full 24 h dataset for those individuals who had it. The *x* axis is the time of year in months, the *y*-axis is the normalized frequency between the two signals, and the color represents the strength of the correlation (yellow high, dark blue low, scale on right hand side). The kind of phase relationship between the signals are noted by the arrows (refer to arrow key).

Since sexual-related behaviors can be expressed differently between sexes, and at least one sex came out significant for both the sexual-related and stereotypic/abnormal behaviors, we displayed the circadian rhythms of these behaviors by sex (Figure 7). The circadian rhythms of sexual-related behaviors were different between the sexes, with most individuals concentrating sexual-related behaviors to daylight hours, but females also showing larger peaks in the night. However, it should be noted that these peaks were displayed by sub-adult females, and therefore, it is unknown if these are patterns true to all females, or a sign of sexual immaturity. In contrast, the circadian rhythm for stereotypic behaviors were quite similar between the sexes, with both sexes displaying a relatively high peak in the morning and smaller peaks throughout the day, without many stereotypic behaviors in the night hours. Interestingly, although the circadian rhythms for these two behaviors were not similar, the circannual rhythms followed a very similar pattern (Figure 8A). Sexual-related behaviors are quite rare, so although the scale is nearly 10× smaller than that of stereotypic/abnormal behaviors, these two behaviors seem to fluctuate in-synchrony throughout the year.

### 3.5. Wavelet coherence for sexual-related and stereotypic/abnormal behavior

To extrapolate more information about this relationship between sexual-related behavior and stereotypic/abnormal behavior, we conducted a wavelet coherence analysis for the two behavior categories (Figure 8B). We found that between the coinciding peaks of these two behavior categories, there was coherence. The two behavior signals were in phase with each other between January and March around the 4 h and 1-day cycle lengths. Around July and October, when both signals were increasing after dipping, there was coherence at the 4 h cycle length with the stereotypic/abnormal signal leading. Then, between November and December, when both signals were fading, there was also coherence at the 4 h and 1-day cycle lengths with the stereotypic/abnormal signal leading.

**Figure 9:**
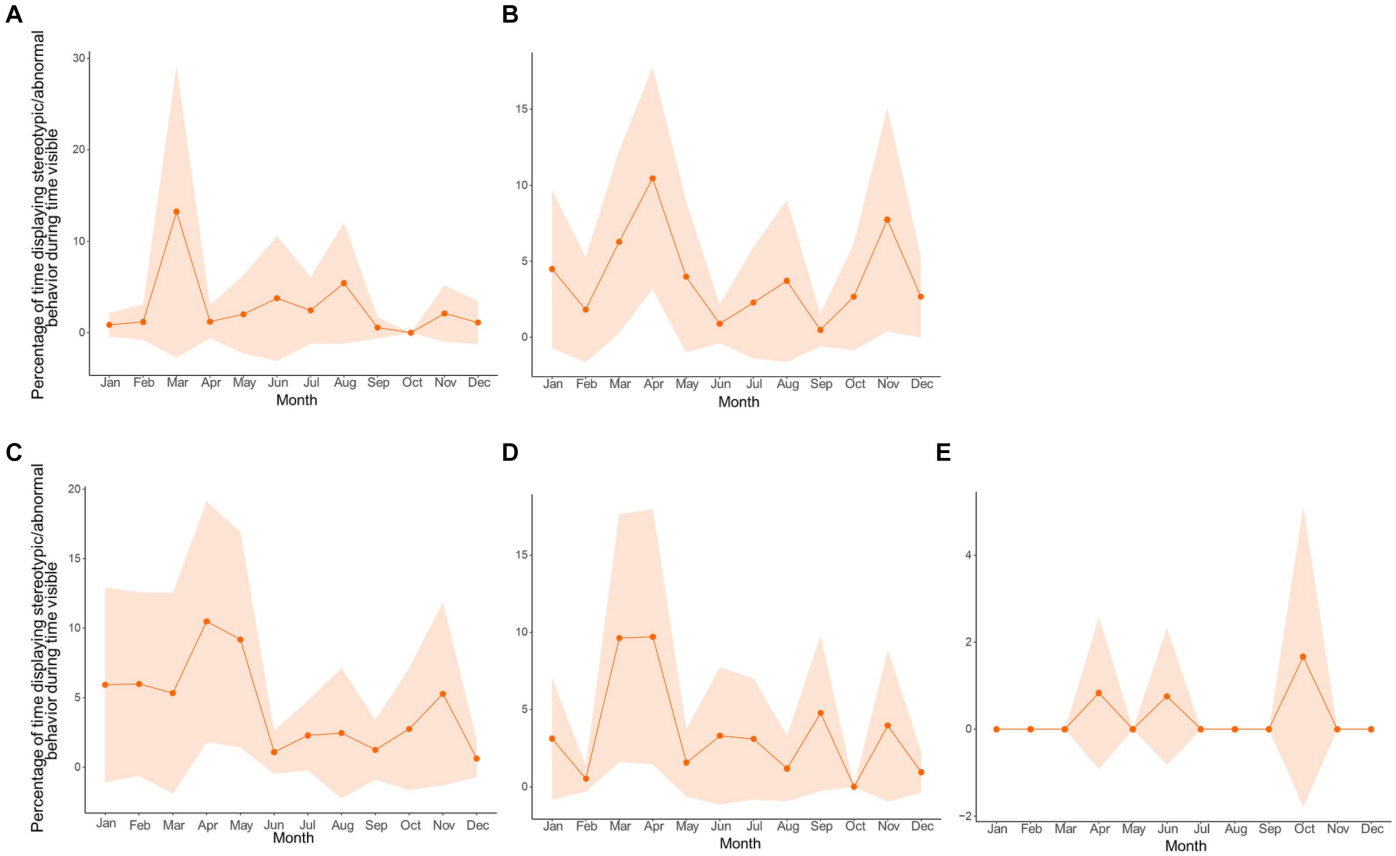
Circannual cycles of the percentage of time in sight displaying stereotypic/abnormal behavior by zoo (lettering following Table 1) with data averaged by month. **(A,C)** are zoos at matched latitudes. **(B,D)** are zoos at mismatched latitudes. **(E)** is a castrated individual (medical reasons) at a mismatched latitude. The shaded regions are 95% confidence intervals.

Since a relationship between stereotypic/abnormal behaviors and sexual-related behaviors was established, and the motivation to display sexual-related behaviors is presumably tied to opportunities to mate, which vary among zoos, we examined the circannual cycles of stereotypic/abnormal behaviors by zoo in line plots (Figure 9). Interestingly, all pandas except for the castrated male in Zoo E, regardless of sexual maturity or mating opportunity, displayed the highest peak in stereotypic/abnormal behavior in the Spring during the mating season. This reinforces the notion that the display of stereotypic/abnormal behaviors may be linked to hormonal fluctuations for breeding, though a larger sample size, especially of castrated individuals, and hormone analyses would still be more informative.

## 4. Discussion

From our results, we found clear relationships and coherence between circadian/circannual cycles and zeitgebers, both natural and zoo specific. Our hypothesis that behavior cycles will change depending on the latitude match/mismatch and the corresponding zeitgebers was supported by the differences we found by latitude for activity and stereotypic cycles. Our second hypothesis that zoos will have additional zeitgebers that animals entrain to was also supported by the differences in indoor/outdoor activity budgets and the peak of activity and stereotypic behavior in the early morning hours that could potentially indicate anticipatory behavior for keepers and fresh food arriving. We have also demonstrated how circadian and circannual cycles can be used to provide a fuller picture of behaviors and their potential regulators, allowing zoos to better manage them and promote positive behaviors by providing a conducive environment and husbandry schedules.

### 4.1. Differences in latitudinal zeitgebers influence activity cycles

Our data on activity showed clear relationships with the external zeitgebers of daylight, temperature minimum, and temperature range, with some differences in coherence and phase relationships between the latitudes. There was also a general finding that pandas at mismatched latitudes were more likely to display inactivity in comparison to pandas at matched latitudes. The difference in the cycles and levels of activity were expected because the zeitgeber cycles themselves differed between matched and mismatched latitudes. Therefore, if animals entrain their external zeitgebers, we expect the activity cycles to shift in accordance with the changes in those zeitgeber cycles.

Despite this, when looking at the averaged circadian cycles of activity between matched and mismatched latitudes, there were no clear differences. The circadian rhythm is an endogenous rhythm that persists in constant conditions (i.e., constant light and temperature; de Mairan, 1729; Golombek and Rosenstein, 2010). Since endogenous clocks are ~24 h (slightly longer or shorter), when allowed to free-run (i.e., under constant conditions), the cycle will drift or display phase delays or advances. Therefore, organisms need daily entrainment to external zeitgebers to be exactly 24 h (Froy, 2011). The pandas displaying similar circadian rhythms despite being at different latitudes with varying photic and non-photic zeitgebers, demonstrates how strong this endogenous cycle can be. What may be occurring is that the circadian cycle of activity will follow a phase pattern throughout the year, but simply needs to be entrained daily to be exactly 24 h. What might shift as the natural zeitgeber fluctuates throughout the year is the amplitude of that phase pattern, not the phase pattern of the circadian cycle itself.

This was demonstrated in the circannual rhythm of activity as the activity levels shifted through the year. Between pandas at matched and mismatched latitudes, the circannual rhythms of activity remained very similar between the groups through most of the year but diverged between December and March. This finding, along with our wavelet coherence analyses between activity and daylight, temperature minimum, and temperature range (Figure 3) show how entrainment might change depending on the rhythm of the external zeitgeber. Changes in annual activity levels for species are triggered by a shift in an external cycle. The external zeitgeber must reach a certain threshold in its signal that is recognized by the animal’s internal clock as an indication of the time of year (e.g., daylight reducing to 6 h as an indication of Winter) and thus trigger the activity appropriate for that time of year. This is seen across species for behaviors like torpor and hibernation, which are induced by changes in food availability, ambient temperature, and photoperiod (Körtner and Geiser, 2000; Genin and Perret, 2003), or migration in birds which is triggered by photoperiods of specific length depending on the season (Gwinner, 1996). If the external zeitgeber is not shifting to the proper threshold, it will not trigger the coinciding shift in activity.

The discussion of potential zeitgebers that influence giant panda activity would not be complete without an understanding of their adaptation to bamboo as a food source. Giant pandas are peculiar in that they have a very low energy expenditure relative to other mammals (Nie et al., 2015). This is a trait that is likely to have evolved because of the inability to digest bamboo fully, to extract the most nutrients. Therefore, pandas must eat large amounts of bamboo to compensate, spending higher proportions of time foraging and feeding than other bear species. We found that pandas spend the majority (70.6%) of their active time feeding. Understanding their feeding ecology is important because their migration patterns have been investigated in the wild, and evidence suggests that their main motivation for migration is to follow the emergence of nutritious bamboo shoots, though there are also effects of solar radiation and habitat preference (Liu et al., 2002, 2011; Zhang et al., 2015, 2017, 2018). Wild pandas initiate migration from mid-April to early June, migrating within several days to their Summer range and returning over several weeks from early September to October (Liu et al., 2002). The fast migration at the start of the Spring coincides with the emergence of bamboo shoots across an elevational gradient, with shoots at higher elevations showing a gradient delay in emergence (Zhang et al., 2018). The ecological factors influencing migration could mean that although we discuss the effects of daylight and temperature on activity below, food availability may also be a zeitgeber in the wild that affects activity and migratory behavior in particular.

Importantly, the initial period of migration also coincides with the breeding season in the Spring. Zhang et al. (2018) used GPS collars and were also able to note that despite the elevational migration pattern across the pandas, the individual paths were distinct, and they were associated with the possibility that pandas were also seeking mates. The synchrony between migratory and breeding behaviors could be linked to the high protein and high amino acid quality of shoots resulting in weight gain (Wang et al., 2017) potentially being beneficial leading into the breeding season, though the increase in culm consumption (plant part with highest caloric absorption) during the summer is still not well understood (Hansen et al., 2010). Ultimately, pandas show seasonal preferences for plant parts in the wild (Liu et al., 2002; Zhang et al., 2015, 2018) and captivity (Hansen et al., 2010; Wang et al., 2017), and with food availability being a potentially strong zeitgeber for migration, further investigation beyond this study would need to be done to understand its potential as a zeitgeber.

#### 4.1.1. Activity and daylight

In our coherence analysis between activity and daylight, the strongest zeitgeber for mammals (Golombek and Rosenstein, 2010), we saw similar coherence and phase relationships between the latitude groups between March and June, but a seemingly missing coherence between August and October for those pandas at mismatched latitudes when compared to pandas at matched latitudes.

The known migratory activity in March/April coincides with the coherence we see in the wavelet coherence for activity and daylight for pandas in both latitudinal locations. This can indicate that daylight may be one of the external zeitgebers that trigger this migratory behavior. The difference we see in the coherence for pandas at mismatched latitudes between August and October around the period of returning migration could be because the daylight cue in the Autumn is not at the proper threshold. The amount of daylight at matched and mismatched latitudes are nearly identical in March and September, however, between October and February, the amount of daylight at mismatched latitudes is much less than at matched latitudes, and much more between April and August. With daylight being a strong indicator of time of year, it may be that pandas at mismatched latitudes receive a daylight signal in the Autumn that is not recognized by their internal clock, or does not reach the proper threshold, and therefore does not trigger a shift in activity. This may be why we see activity levels between latitudes diverge between November and April. These differences in activity levels and synchronization with the daylight zeitgeber between the latitude locations does not necessarily indicate poor welfare for either location, but supports the argument that animals in a captive environment will respond to external cues with shifts in their activity. Therefore, this should be known by staff who house these animals, so they can be aware of when to expect changes in activity and how to modify the environment accordingly. For migratory species, allowing access to more or larger areas during periods of natural migration may be a solution.

#### 4.1.2. Activity and temperature

Though temperature is a weak zeitgeber for most endotherms, it still plays an important role in resetting circadian oscillators and enhancing internal circadian synchronization (Buhr et al., 2010). Refinetti (2010) found that mice respond to temperature cycles as a zeitgeber, though not as efficiently as they do to daylight. These findings indicate that although temperature is not as strong a zeitgeber as daylight, it is still important to consider how it may influence the behavioral cycles of a captive animal. Indeed, in our study, we did find a relationship between activity and temperature measures with activity decreasing as both temperature minimum and temperature range increase. Previous research on pandas also suggests that temperature is an important external cue for activity. Liu et al. (2011) investigated solar radiation in relation to the monthly distribution of pandas in the Foping Nature Reserve. Solar radiation is the intensity of sunlight and therefore directly related to temperature, with higher solar radiation resulting in higher temperatures. They found that pandas begin migrating in Spring when the solar radiation changes from low to high and migrate back when the radiation shifts from high to low, indicating a potential regulatory effect of solar radiation on migratory patterns. They also found that the panda tracking locations had a much higher minimum solar radiation level than the minimum solar radiation for the whole of the nature reserve. This indicates that the pandas use solar radiation minimums to select their habitat. Zhang et al. (2015) expanded on this finding and discovered that pandas choose areas with lower solar radiation in warmer months and higher solar radiation in cooler months (Zhang et al., 2015). In addition, Zhang et al. (2017) conducted wavelet coherence analyses between activity and solar radiation and temperature. They found coherence with activity at the 1-day cycle length throughout much of the year for both solar radiation and temperature, indicating that pandas synchronize their daily activity cycles with daily temperature cycles.

Similarly, in our coherence analyses with temperature, we found coherence between temperature minimum and activity at the 1-day cycle length during warmer months for pandas at matched latitudes but not mismatched latitudes. This would be the season in which the temperature minimum reaches its highest point at both latitude locations. However, in the mismatched latitudes, the temperature minimum in summer was reaching temperatures similar to temperature minimums in Spring and Autumn for the matched latitudes. Therefore, it could be that this is another cue that does not reach the proper threshold in mismatched latitudes to synchronize with activity levels. However, despite the absence of coherence between temperature minimum and activity during warmer months for pandas at mismatched latitudes, activity levels were similar for summer between the latitude groups. Interestingly, for the temperature range and activity coherence, we found coherence at the 1-day cycle length in summer for pandas at mismatched latitudes but not at matched latitudes. The temperature ranges between the latitude locations were similar from May–September and quite dissimilar from October–April. Therefore, further investigation could determine if during warmer months, pandas will initially synchronize their activity levels to temperature minimums, but if that cue is not reaching the proper threshold, they will use temperature range as a similar cue. It would also be beneficial to investigate how temperature affects their choice in habitat in a captive environment, and whether captive pandas, like their wild counterparts, will choose cooler areas in the summer and warmer areas in the winter. In a zoo environment, this habitat selection can be the choice between an indoor and outdoor enclosure. Understanding these relationships between activity, temperature, and habitat choice can aid zoos in determining appropriate temperature conditions so that they can provide the proper choices at the right times of year and promote circadian synchrony.

### 4.2. Zoo specific zeitgebers can potentially entrain behavior cycles

With temperature and lighting being clear zeitgebers that regulate activity, it was important to investigate how the climate controlled indoor enclosures in zoos influenced activity. We found that the circadian cycle of activity fluctuated in synchrony with the percentage of time spent outdoors. This can be due to several factors. One explanation may be that pandas prefer to rest indoors where the climate is controlled. Another explanation may have to do with the fact keepers will often move food to the outdoors to lure the pandas outdoors so that they may clean their indoor enclosures. This means that they would often be feeding (the most common active behavior) while outdoors. To investigate further, we looked at activity budgets indoors and outdoors and examined the latitudinal differences since pandas at mismatched latitudes would presumably be experiencing natural zeitgebers outdoors that they may not be adapted to. Pandas at matched latitudes had very similar indoor/outdoor activity budgets, while pandas at mismatched showed a significantly higher proportion of locomotive activity outdoors.

The cause of the difference in locomotor activity between indoor and outdoor enclosures in pandas at mismatched latitudes but not matched latitudes could not be determined but may be due to preferences for increasing locomotion in colder temperatures (outdoor temperatures at mismatched latitudes toward the poles would be cooler) to decrease energy expenditure (Nie et al., 2015) or due to enclosure design. Further investigation with larger sample sizes and clearer information on schedules for when pandas have access to indoor/outdoor enclosures would be needed to determine if differences in temperatures and lighting have significant effects on activity budgets. However, our findings do provide some evidence that the climate control for indoor enclosures at mismatched latitudes expose the animals to a different set of zeitgebers than those experienced in outdoor enclosures. As mentioned in the discussion on temperature minimum, if a captive animal chooses their habitat based on temperature, then it would be ideal for zoos to understand the temperatures they prefer at different times of year and ensure that they do provide their animal with these choices at the appropriate times of year within the indoor enclosures. Zoos often attempt to mimic the specific species’ natural climate conditions within their indoor enclosures. However, if this cannot be done, it may be easier to house animals within their natural latitudinal range. Zoos may also want to consider whether only an indoor enclosure with appropriate conditions is adequate for their animal, or if an outdoor area with zeitgebers and climate they are evolved for is also needed.

Aside from the added zeitgebers of the controlled climates of indoor enclosures, husbandry routines which include feeding, cleaning, and training sessions become a part of the external environment of the animal that is predictable (Bassett and Buchanan-Smith, 2007) in the same way light and temperature are, and cause arousal, making them a potential non-photic cue. Therefore, in response to husbandry practices, animals may show predictable changes in their behavior and physiology. Our results may indicate some form of anticipatory behavior for feeding or husbandry practices since there is a clear peak of activity in the early morning hours when keepers arrive. Pandas across all the zoos studied were left with bamboo overnight and would receive fresh bamboo in the mornings when keepers arrived. This prediction of anticipatory behavior could be further supported by the same peak in stereotypic/abnormal behavior in the early morning-which does not coincide with the circadian cycle of sexual-related behaviors- because predictable feeding schedules also cause animals to display anticipatory behaviors which may signal stress should their frequency increase in response to the predictable schedule becoming delayed (Waitt and Buchanan-Smith, 2001; Anderson et al., 2015).

### 4.3. Determining circadian and circannual cycles of wanted and unwanted behaviors can help zoos manage them

Stereotypic/abnormal and sexual-related behaviors are of major concern for zoos, with the former behaviors being unwanted and the latter being crucial to the goal of conservation of species. Therefore, understanding these behaviors and the factors that may regulate them are of extreme importance to zoo staff and have many implications for the goals of zoos. Our findings on the relationship between sexual-related and stereotypic/abnormal behaviors in giant pandas provided further evidence on the previously determined relationship by Martin et al. (2020). Their results found seasonal differences in the amount of stereotypic behavior displayed along with sex differences across those seasons. Our results support the findings that there is a strong relationship between sexual-related behavior and stereotypic/abnormal behavior. We also have similar results indicating that stereotypic/abnormal behavior and sexual-related behavior vary across seasons, with pandas showing significantly less stereotypic/abnormal behaviors in the Winter, and increased sexual-related behavior in the Spring, as expected with the breeding season. However, we did not find sex differences in the amount of stereotypic behaviors displayed.

Our results were able to illuminate how this relationship between sexual-related and stereotypic behavior is expressed. On a daily scale, the cycles of stereotypic/abnormal and sexual-related behaviors do not synchronize. However, on an annual scale, the cycles do fluctuate in synchrony. The circadian cycle of sexual-related behavior shows adults concentrating their sexual-related behavior very efficiently to daylight hours, while sub-adults display these behaviors more randomly throughout the day/night. These age group differences could potentially indicate that the time of day in which sexual-related behavior is displayed is a mark of sexual maturity. There may be a benefit to concentrating sexual-related behaviors to daylight hours so that mating pairs are synchronized in their behavior, increasing the chances of successful mating. Synchrony of reproductive behaviors between reproductive individuals is an adaptive benefit to having a circadian rhythm. For instance, in nocturnal rodents, ovulation occurs at night when encountering a mate is most likely (Goldman, 1999). For animals with a reproductive season, monitoring the time of year *via* the circadian clock (i.e., measuring day length) allows the animal to ovulate when environmental conditions are most favorable for gestation and rearing (Boden and Kennaway, 2006). Further investigation would need to be done to determine if this concentration of sexual-related behavior to daylight hours is regulated by hormonal cycles that develop when a giant panda reaches sexual maturity. However, the information on the behavioral cycles alone can help zoos understand the optimal time of day to provide their pandas with mating opportunities.

The synchronized fluctuation and coherence between the circannual cycles of sexual-related and stereotypic/abnormal behaviors was a very informative finding. The wavelet coherence displayed that the stereotypic/abnormal signal are in phase around the breeding season, and outside of the breeding season, the stereotypic/abnormal signal leads. These phase relationships may indicate that captive pandas express stereotypic behaviors out of frustration for not being able to express sexual-related behavior, displaying an increase in stereotypic/abnormal behaviors with sexual-related behaviors as a way to fulfill the unmet needs. This prediction is further supported by the fact that for our study pandas, the most common stereotypical behavior was pacing. This makes sense with it being related to sexual behaviors because pandas migrate during the mating season, with wild males showing increased locomotion in the mating season when compared to females (Liu et al., 2002), and captive males showing more locomotive stereotypies than females and the behavior correlating with reproductive performance (Martin et al., 2020). Also, our study pandas tended to incorporate anogenital rubbing and handstand urination into their pacing or bipedal standing. Anogenital rubbing and handstand urination are sexual-related behaviors because scent marking is used as a chemical signal of home range occupation and potentially fitness, with males scent marking more often than females (White et al., 2002; Charlton et al., 2020). Bipedal standing (done at keeper doors) would be related to anticipatory behavior with keepers as mentioned before.

Further evidence of this relationship between stereotypic/abnormal and sexual-related behaviors can be seen from our finding that pandas at mismatched latitudes are less likely to display any level of stereotypic/abnormal behavior when compared to pandas at matched latitudes. Since Spring migration and the breeding season occur at the same time, and we found that daylight is a potentially strong zeitgeber that synchronizes with activity for pandas at matched latitudes at the same times of year that they are likely to be migrating back and forth, we would want to investigate further whether the same synchrony that is missing in the mismatched latitudes would also be a missed threshold for mating behavior in mismatched latitudes. Although our study did not find any relationship between daylight, temperature minimum or temperature range with sexual-related behaviors, the behaviors are extremely rare and therefore a larger sample size with higher resolution signals would help us explore the potential zeitgebers for sexual-related behavior. However, since sexual-related and stereotypic/abnormal behaviors are related, and temperature range showed a significant relationship with activity, and a trend toward significance with stereotypic/abnormal, this cue could merit further investigation. Interestingly, for two of the zoos, temperature range fluctuated in synchrony with stereotypic/abnormal behavior. In addition, though only a sample of one, there was an interesting result where the castrated individual displayed the least, and almost no amount of stereotypic/abnormal behaviors. This could support the theory that stereotypic/abnormal behaviors are instigated by an unmet desire to display sexual-related behaviors, so when that desire is reduced from castration, so are the levels of stereotypic/abnormal behavior.

Our results elucidating the relationship between sexual-related and stereotypic/abnormal behaviors is a demonstration of how zoos can use information about circadian and circannual cycles of behavior to understand and manage these behaviors. Although this relationship cannot be generalized to all species, the manner in which we determined the relationship and understood it can be extended to other species to determine the triggers for stereotypic/abnormal behaviors so that they may be reduced, and to promote sexual-related behaviors. For giant pandas, the expression of stereotypic/abnormal behavior with anogenital rubbing and handstand urination can be an indication to keepers that the pandas would like a mating opportunity. In addition, since sexual-related and stereotypic/abnormal behavior is displayed approximately every 3–4 months in our study pandas, we would recommend that zoos consider allowing mating opportunities outside of the breeding season because they may actually mate at different times of year if allowed.

## 5. Conclusion

Given the large influence that circadian rhythms have on behavioral and physiological processes, it is essential that within a captive environment, they are well understood across species. The welfare of a species can be difficult to assess if all the factors that influence their welfare are poorly understood or analyzed separately. By assessing rhythmicity across the day, night, and seasons, we are contextualizing behavioral responses on a broader scale and understanding both internal and external factors which may influence their behavior and ultimately welfare. Individual organisms do not exist in a vacuum of conditions, their continued existence depends on their ability to properly synchronize their internal environment with the external one in order to anticipate changes and respond accordingly through physiological or behavioral changes (Golombek and Rosenstein, 2010). The optimal external conditions for an organism to live in are the conditions for which their circadian rhythm has evolved. Therefore, in captive environments, we want to attempt to provide these optimal conditions to our animals as well as understand how conditions which they would not experience in the wild can affect them at individual, group and species level.

Through this study we have demonstrated a holistic approach to identify complex interactions between an animal and their external environment. Zoos can take this approach of systematically monitoring behavior, even if slowly over time, to understand their animals’ needs and apply the information to construct appropriate enclosures and husbandry practices/schedules, taking into account how those needs change cyclically over time. As a community, zoos can also use this approach and information to conduct further research to address questions at the forefront of captive animal welfare on whether species should be housed outside of their natural climatic and latitudinal conditions. Our results demonstrate that there are clear relationships between the cycles of external factors and behavior. Therefore, it would benefit zoos to investigate these relationships further so that they may provide their animals with appropriate choices, enhance conservation efforts, promote circadian synchronicity, and consequently improve welfare.

## Data availability statement

The datasets presented in this study can be found in online repositories. The names of the repository/repositories and accession number(s) can be found at: http://hdl.handle.net/11667/208.

## Ethics statement

The animal studies were approved by the University of Stirling Animal Welfare and Ethical Review Body. The studies were conducted in accordance with the local legislation and institutional requirements. Written informed consent was obtained from the zoos housing the giant pandas for the participation of their animals in this study.

## Author contributions

KG, SK, and HB-S contributed to conception and design of the study. KG collected the data, organized the database, performed the statistical analysis, and wrote the first draft of the manuscript. SK and HB-S restructured and edited sections of the manuscript. All authors contributed to manuscript revision, read, and approved the submitted version.

## Conflict of interest

The authors declare that the research was conducted in the absence of any commercial or financial relationships that could be construed as a potential conflict of interest.

## Publisher’s note

All claims expressed in this article are solely those of the authors and do not necessarily represent those of their affiliated organizations, or those of the publisher, the editors and the reviewers. Any product that may be evaluated in this article, or claim that may be made by its manufacturer, is not guaranteed or endorsed by the publisher.
