# Supplementary material for: Latitudinal and zoo specific zeitgebers influence circadian and circannual rhythmicity of behavior in captive giant pandas (Ailuropoda melanoleuca)
Source: Front Psychol. 2023 Sep 18;14:1188566. doi: 10.3389/fpsyg.2023.1188566 (PMC10543212; doi:10.3389/fpsyg.2023.1188566)
Supplement: Supplementary file 1 [file Table_1.docx]

Supplementary Table 1. Panda ethogram used to code behavior, and references from the literature from which the definitions were adapted. Our interpretation of welfare valence is when the giant panda performed the behavior at its species’ typical levels for captive individuals. Reproduced from Gandia et al. (2023).

| Behavior | Welfare valence | Description | Reference |
| --- | --- | --- | --- |
| Solitary | | | |
| Resting/  Sleeping | Neutral | Inactivity (lying, sitting or standing on all four), either awake or asleep. If standing, the panda should not be investigating or seem as though attention is on anything specific. Shifting between resting positions still counts as resting as long as it does not involve locomotion or becoming alert. | (Ryan and Litchfield 2020) |
| Locomotion | Neutral | Directional travel at any speed. Only includes short bouts of travel with a seemingly intended goal or long bouts of travel with no evidence of a stereotypic or repetitive pattern. Includes climbing. |  |
| Feeding/  Foraging | Positive | The manipulation or eating of food/water provided by keepers or foraging for plants growing in enclosure. Includes food search activity, moving around enclosure, sniffing ground or air. |  |
| Play | Positive | Playful running, gymnastics, interacting with objects (paw or mouth manipulation of objects – includes enrichment items). Includes playing in water (for example splashing). |  |
| Investigate | Positive | Panda places nose within 5 cm of inedible object and sniffs or licks the object. | (Swaisgood et al. 2001) |
| Anogenital rubbing (sexual-related) | Positive | Applying pressure to the hind area below the tail by moving the hind quarters in a back-and-forth motion around or up and down on the surface of an object or on the wall. Can be performed in four positions: squat, reverse (backing up to a vertical surface), leg cock (against a vertical surface with one leg raised), and a full handstand (both hindfeet elevated off the ground with body fully extended). | (Liu et al. 2005, White, Swaisgood, and Zhang 2002) |
| Scent anointing (sexual-related) | Positive | Rubbing face, head, neck, and/or shoulders on object or wall in fluid movements or by rolling on it, making contact with head, neck, shoulders, and upper back. Can use paws to try and spread odor. | (Bian et al. 2013, Charlton et al. 2020) |
| Urinating/  Defecating | Neutral | Assuming a squat, leg-cock, handstand, or standing posture on the wall or ground to excrete urine or feces. | (Liu et al. 2005) |
| Grooming | Positive | Scratching and licking of the pelage. |  |
| Drinking | Neutral | Placing mouth at surface of pools or spouts and ingesting water. |  |
| Keeper interaction | Depends | Training or any interaction where keeper is intentionally trying to gain and maintain the attention of the panda. Can occur at any location in enclosure where keeper is visible to the panda. |  |
| Social | | | |
| Aggressive | Negative | Animal forcefully swats with forepaws, lunges towards other individual, grapples, or bites with force. | (Owen et al. 2013) |
| Show interest (sexual-related) | Positive | Animal responds to the other party by sniffing the other participant, pushing or pulling at the fence between, or swaying or locomoting back and forth with proximity of the other animal which must be clearly in view, even if through a mesh barrier. |  |
| Sexual (sexual-related) | Positive | Female presents anogenital region to male. Male sniffs or licks anogenital region and/or nudges or paws at anogenital region through wire mesh. Note that the female must first display “sexual” behavior before the male responds with “sexual” behavior. |  |
| Social play | Positive | Nonaggressive chasing, wrestling, inhibited biting or pawing at other individual. There should not be attempts to escape and individuals can alternate between subordinate and dominant positions. |  |
| Stereotypic/Abnormal | | | |
| Pace | Negative | Stereotypic pacing (back and forth, or perimeter locomotion, in a repetitive sustained pattern, tracing the same route at least 3 times consecutively) or quasi-stereotypic pacing (same as stereotypic pacing, except animal need not take the same path 3 or more times in a row. Any pacing in which a predictable pattern emerges. There may be variations in the routine or the animal may alternate between a limited number of travel paths). | Panda project PDX Wildlife* |
| Bipedal standing | Negative | Standing on hind legs and looking through glass, fence, or outside enclosure seemingly in anticipation of something. | (Liu et al. 2003) |
| Self-mutilation | Negative | Self-inflicted physical harm, such as biting or chewing the tail or leg, or hitting the head against a wall. |  |
| Cage climb | Negative | Stands bipedally and sways or makes climbing motions, as if attempting to escape. | (Swaisgood et al. 2005) |
| Regurgitation | Negative | Vomits and reingests vomit repeatedly. |  |
| Pirouette | Negative | Stands on hind legs and spins body at least 90 degrees. |  |
| Head-toss | Negative | Swings upward or to the side in a swinging movement. |  |

* <https://www.pdxwildlife.com/become-a-citizen-scientist/> (accessed 1 May 2020)

References

Bian, X., D. Liu, H. Zeng, G. Zhang, R. Wei, and R. Hou. 2013. "Exposure to odors of rivals enhances sexual motivation in male giant pandas." PLoS One 8 (8):e69889. doi: 10.1371/journal.pone.0069889.

Charlton, Benjamin D, Megan A Owen, H Zhang, and Ronald R Swaisgood. 2020. "Scent anointing in mammals: functional and motivational insights from giant pandas." Journal of Mammalogy 101 (2):582-588.

Gandia, Kristine M, Elizabeth S Herrelko, Sharon E Kessler, and Hannah M Buchanan-Smith. 2023. "Understanding Circadian and Circannual Behavioral Cycles of Captive Giant Pandas (Ailuropoda melanoleuca) Can Help to Promote Good Welfare." Animals 13 (15):2401.

Liu, Dingzhen, Zhipeng Wang, Hong Tian, Changqing Yu, Guiquan Zhang, Rongping Wei, and Heming Zhang. 2003. "Behavior of giant pandas (Ailuropoda melanoleuca) in captive conditions: Gender differences and enclosure effects." Zoo Biology: Published in Affiliation with the American Zoo and Aquarium Association 22 (1):77-82.

Liu, Dingzhen, Guiquan Zhang, Rongping Wei, Hemin Zhang, Jiming Fang, and Ruyong Sun. 2005. "Behavioral responsiveness of captive giant pandas (Ailuropoda melanoleuca) to substrate odors from conspecifics of the opposite sex." Chemical Signals in Vertebrates 10.

Owen, Megan A, Ronald R Swaisgood, Laura McGeehan, Xiaoping Zhou, and Donald G Lindburg. 2013. "Dynamics of male–female multimodal signaling behavior across the estrous cycle in giant pandas (Ailuropoda melanoleuca)." Ethology 119 (10):869-880.

Ryan, Jillian Claire, and Carla A Litchfield. 2020. "Impact of an enclosure rotation on the activity budgets of two captive giant pandas: An observational case study." Eat, Sleep, Work 1 (1):26-38.

Swaisgood, Ronald R, Angela M White, Xiaoping Zhou, Guiquan Zhang, and Donald G Lindburg. 2005. "How do giant pandas (Ailuropoda melanoleuca) respond to varying properties of enrichments? A comparison of behavioral profiles among five enrichment items." Journal of Comparative Psychology 119 (3):325.

Swaisgood, Ronald R, Angela M White, Xiaoping Zhou, Hemin Zhang, Guiquan Zhang, Rongping Wei, Valerie J Hare, Erin M Tepper, and Donald G Lindburg. 2001. "A quantitative assessment of the efficacy of an environmental enrichment programme for giant pandas." Animal Behaviour 61 (2):447-457.

White, Angela M., Ronald R. Swaisgood, and Hemin Zhang. 2002. "The highs and lows of chemical communication in giant pandas (Ailuropoda melanoleuca): effect of scent deposition height on signal discrimination." Behavioral Ecology and Sociobiology 51 (6):519-529. doi: 10.1007/s00265-002-0473-3.
